# The coronavirus response: Boxed in by models

**DOI:** 10.1177/1356389020968579

**Published:** 2020-11-05

**Authors:** Ray Pawson

**Affiliations:** University of Leeds, UK

**Keywords:** complex systems, coronavirus, infectious disease modelling, non-pharmaceutical interventions

## Abstract

Science has a mixed record when it comes to predicting the future. Engineers build bridges based on foreknowledge of the forces that they are likely to encounter – and their constructions tend to withstand the test of time. Predicting the future course of epidemics and building intervention to contain them are much more precarious. And yet simulation models produced in prestigious centres for mathematical biology have played a significant role informing coronavirus policy in the United Kingdom and elsewhere. The predictive uncertainties include the inherent variability of the pathogen, considerable variation in host population immunity as well as the concern of this article, namely, the constantly adapting human judgements of those designing, implementing and experiencing the national response to an outbreak. Assumptions about how interventions are implemented and how people will react are, of course, built into modelling scenarios – but these estimates depict behavioural change in fixed, stimulus-response terms. Real reactions to the complex restrictions introduced to combat the virus unfold in scores of different pathways – people comply, they resist, they learn, they grow weary, they change their minds, they seek exceptions and so on. Model building is intrinsically speculative, and it is important that crisis management is not boxed in by its latent simplifications. A more pluralistic evidence base needs to be drawn on, to understand how complex interventions operate within complex societies.

On two occasions I have been asked, ‘Pray, Mr. Babbage, if you put into the machine wrong figures, will the right answers come out?’ . . . I am not able rightly to apprehend the kind of confusion of ideas that could provoke such a question.

– Charles Babbage, the Father of Computing (1864)

## Introduction: All models are wrong?

‘All models are wrong’. Not many methodological maxims have their own Wikipedia entry, but this striking aphorism, usually attributed to the British statistician George Box, takes on particular significance as science struggles to predict the trajectory of the coronavirus outbreak. In the face of the pitiless challenge of the COVID-19 pandemic, political leaders throughout the world have been quick to vouchsafe that they will ‘follow the science’. The science in question, mathematical biology, was perhaps not so well known to the public, but in the space of a few months, the basic concepts and imagery associated with the modelling of infectious disease have become remarkably familiar. Not a day goes by without a bulletin from the Assemblée nationale or from the Bundestag or from Westminster about renewed efforts to ‘flatten the curve’ or about the latest value of the ‘*R* number’. Modelling terminology has taken on emblematic significance. But what exactly does it foretell? And how seriously should we take Box’s doleful warning?

The article tackles these questions in three sections. The first outlines the basic methodology of infectious disease modelling. It explains the construction of the disease transmission curves that carry the predictions and extrapolations of epidemic models, in particular, the underlying statistical assumptions about how social intervention gain traction is scrutinised. The second section examines how modern programme evaluation answers these same questions. There is a vivid contrast insofar as social interventions are understood by evaluation as complex, adaptive, self-transforming systems, which go on to produce complex, varied and often unanticipated outcomes. The third section portrays the resultant clash. It provides detailed examples from two prominent UK simulations of the considerable gap between mechanical assumptions of human behaviour portrayed in ‘model world’ and the mercurial responses to the virus played out in the ‘real world’. There is a short conclusion.

## Epidemic modelling

**Figure 1.**
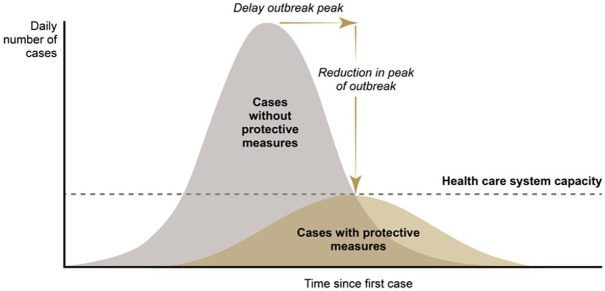
Modelling scenarios: Flattening and delaying an outbreak.

Infectious disease models use a standard format for presenting predictions on the likely course of the outbreak, as illustrated in Figure 1. It takes the form of two transmission curves. The first portrays what is considered the ‘natural’ course of the disease and is often labelled ‘cases without protective measures’ (known colloquially as the ‘do nothing’ curve). The number of cases, which may be measured as infections or deaths or hospital admissions, is estimated on the vertical axis. The progress of the disease through the population is estimated on the time axis. The ultimate goal is to understand the momentum of the virus and thus help to prevent the swamping of hospital and emergency care provision, to which end estimates of healthcare capacity are customarily included in the graphics, as shown.

How is disease progression modelled? The natural transmission curve in a major epidemic takes shape as a disease moves through four groups – the susceptible, the infected, the recovered and the deceased. Epidemiology makes use of a generic pattern, the bell shape, whereby the number of cases increases exponentially until the proportion of the susceptible has been sufficiently depleted (through recovery or death) so that the growth rate then slows and the number of cases eventually drops so that the epidemic is no longer sustained (Keeling and Danon, 2009). This normal curve of transmission is based on a theoretical model first produced in 1864 that became known as the SIR model (i.e. susceptible, infected, recovered), which generates the steady progression and remission depicted in Figure 1. This basic model relies on many simplifications, that is, assumptions about the homogeneous mixing of contacts through a population and the contact networks remaining unaffected by the dynamics of the epidemic.

More advanced models, used today, incorporate many more parameters in order to improve upon these assumptions and sharpen the predictions. The first set of these accommodations provides estimates for the basic biological drivers of an outbreak. Best known is ‘basic reproduction number’ of the virus, denoted *R*_0_. This provides a measure of its initial transmission potential, the average number of secondary cases generated by a single infected individual. In popular parlance, it is often understood as an indicator of the basic ‘aggression’ of the infection. It allows us to differentiate routine infection from epidemic – the higher the *R*_0_, the faster the disease will accelerate and the more pressure will be exerted on medical provision (Aronson et al., 2020). Other biological parameters routinely applied in the initial modelling include estimates of the incubation period, levels of natural immunity, transmission mechanisms and pathogen variability (Siettos and Russo, 2013). At initial outbreak, these epidemiological processes are not fully understood, and they have to be approximated, usually on the basis values derived from previous epidemics or from countries first encountering a virus. We will return to the adequacy of these clinical estimates when we examine specific UK models.

Parameterisation, to use the jargon, continues with a further set of predictors, those used to estimate the social circulation of the disease. Transmission lies in human contact and demographics like population size and density, family and age structures, and the size of social networks are examined and estimated. More sophisticated models may include assumptions about work and travel patterns and how these might literally ‘transport’ the disease. Other models subdivide the population into subgroups by region, race, sex, health status, comorbidity and so on in order to estimate the differential reach of the disease. The research effort expended here is enormous (witness the 20/30 authors often listed in publications). The overall strategy, to use a splendidly dramatic summary of epidemic modelling by Adam (2020), is to ‘build a virtual copy of a city, region, or entire country using differential equations to model the interactions of population groups in space and time. They then seed this world with an infection and watch how things unfold’.

We will examine in due course how closely the ‘virtual copies’ match up to the real thing. But first we must complete the basic account of model building and describe how the second curve in Figure 1, the one including the ‘protective measures’, is constructed. This ‘do-something’ curve seeks to predict the impact on disease progression of the mitigation measures that may be put in place to contain the virus. Alas, it is necessary to make clear that the comparison modelled is not a comparison of inaction and real action; it is a comparison of two hypothetical scenarios. The second scenario is manufactured simply by modifying the parameters used in the first. These adjustments may take account of potential advances in immunology and treatment but given the time taken to propel these measures through clinical trials, models have concentrated on the major mitigation effort in the COVID-19 outbreak, namely, ‘non-pharmaceutical interventions’ (NPIs).

These measures came thick and fast as the virus deepened its hold: hand hygiene advice, surface cleaning, provision of protective equipment, recruitment and training of additional staff, building new facilities, the closure of shops, stadiums and schools, social distancing, isolation and lockdowns, working from home, testing and tracing and so on. All of these seek to block the circulation of the virus. They are classic social interventions – instigated via extra spending or legislation, but mostly they are transmitted as government ‘guidance’. The modellers’ task here is to predict the behavioural changes that will follow this medley of measures. How many people will comply with the guidance? What proportion of the population will be taken out of circulation by a particular measure? How will crisis management change social interaction through space and time?

Providing answers to these fundamental questions depends on anticipating of dozens of micro-manoeuvres. How many people, with what institutional support, will self-isolate, work from home, take up the furlough scheme, obey travel restrictions, abandon public transport, trade less, keep to the 2-metre rule, commit to home schooling, modify family ties, establish social bubbles, reduce medical consultations and so on. No one knows the answers to these questions in advance of policy implementation, but estimating the extent and effectiveness of such social processes is precisely the task entrusted to the model builders. Various ‘change scenarios’ are pondered – *x*% of population will comply with measure A; *y*% will comply with measure B and so on. The modified transmission dynamics are then loaded into revised equations, generating the second, ‘protective-measures model’ as in Figure 1. Note again that the percentage estimates of the organisational and behavioural responses may be guided by reviewing the utility of various measures in previous epidemics – but basically, they are approximations. We are left with the ultimate question. Should they be regarded as trusted estimates or are they simply guesswork?

And this brings us back to George Box. Box was a pioneer of statistical model building and so his tongue was partly in cheek when he first used that enigmatic phrase in this form: ‘All models are wrong, but some are useful’ (Box, 1976). Box’s essential idea, developed across a range of further publications, was that the worth (or the inadequacy) of a model depended on the veracity (or the deficiency) of the many assumptions and estimates built into it. His thesis more fully formed goes like this:

All models are approximations. Assumptions, whether implied or clearly stated, are never exactly true. All models are wrong, but some models are useful. So the question you need to ask is not ‘Is the model true?’ (it never is) but ‘Is the model good enough for this particular application?’ (Box and Luceño, 1998)

## Enter complexity: The coronavirus response as a complex, adaptive, self-transforming system

Let us follow this advice and examine in some detail the ‘particular application’ embodied in the UK national policy response to the coronavirus crisis. It has become a cliché to describe the virus and the response as ‘unprecedented’ and it is important to test this utterance. How vast is the challenge? What exactly are we trying to model? One useful way of approaching these questions is to use the lens of complexity theory. In doing so, we change disciplines and we change mind-sets, shifting from ‘modellers’ to ‘evaluators’. Programme evaluation has its own history and its own methodology. Increasingly, it has come to appreciate that public policy is delivered by complex interventions thrust into complex environments.

Complex adaptive systems are part of a ‘turn’ towards complexity and systems thinking across the social and applied sciences that feature increasingly in the evaluation of complex programmes and policies (CECAN, 2018; Gates, 2016; Gerrits and Verweij, 2015). These ideas have even found their way into the *Magenta Book*, formal UK Treasury guidance (HM Treasury, 2020) on what to consider when designing evaluations. Therein the properties of complex systems are laid out in detail (adaptation, emergence, unanticipated consequences, feedback loops, blockage points and structures, non-linearity, tipping points, path dependency, openness and self-transformation). This is no place to explicate these many disorderly processes. It is relatively simple, however, to illustrate that they are deeply rooted in the interventions designed to combat the virus. To this end, Box 1 enters the ‘black box’ of the UK programme. It provides an outline, with very brief examples, of the multiplicity of self-transforming and interacting processes that make up the coronavirus response. It begins to map the countless practical dilemmas faced by researchers attempting to untangle how and how well the epidemic is being controlled.

**Box 1.** UK coronavirus policy: elements and examples.

The response . . .	Examples
Consists of scores of separate interventions (hand hygiene, surface cleaning, protective equipment, closure of shops, stadiums and schools, social distancing, working from home, testing and tracing, etc.) which interact and may compete with and may stymie one another.	Job retention (furlough) schemes reduce transmission but drive deficits. Discharging elderly hospital patients without testing increase care home transmission. Isolation measures increase mental health problems, domestic abuse, educational disadvantage but decrease A&E loads, cancer referrals, pollution levels and so on.
Requires as much if not more attention to ‘exit’ as it does to ‘entry’. Unlocking is significantly more difficult to phase, manage and implement than lockdown.	Closing schools, shops, stadiums and so on is much easier than reopening them with capacity limitations, one-way systems, sanitising points, screening and booking systems. Messaging to ‘stay at home’ is more easily comprehended and actioned than alerting people to ‘stay safe’ and so on.
Involves long implementation chains, which adapt the interventions on their way to the public. Central government or ‘top down’ interventions are continually reinvented by intermediaries over time, generating intended and unintended consequences.	Hospitals and care homes extemporise under demand pressures, PPE shortages and staff absences. Policing policy on unofficial gatherings varies by constabulary. Schools differ in maintaining provision for the at-risk and the children of key workers. Parents disagree about safety levels on reopening. Resolve to isolate and distance weakens over time and so on.
Is deeply contextual, with the same measures generating different outcomes in different communities and countries. Both the transmission potential and the capacity to respond vary significantly from location to location.	Disease prevalence varies significantly by subgroup. The *R* number varies from neighbourhood to neighbourhood. Compliance with guidance varies with national and local culture. Public health discipline changes. Very young children, dementia sufferers and the drunk and disorderly have little capacity to obey distancing rules. Guidance is continually tested by ‘free riders’ and so on.
Is continually buffeted by political dogfights, with frequent changes in strategy and in action plans. The timing of the introduction and withdrawal of specific interventions is influenced daily by media and social media pressures.	UK *Daily Mail* (23 March 2020). ‘The world is responding to coronavirus. Towns in lockdown, mass gatherings banned and increasing border checks to battle the pandemic . . . But Boris Johnson declines to stop UK sporting events and says closing schools could make the crisis WORSE’ and so on.
Consists of a complex, adaptive, self-transformative system, thrust into a complex, adaptive, self-transformative system. All policies, including those directed at epidemics, operate in a wider, recursive cycle of reforms.	Coronavirus interventions shape and are shaped by other contemporaneous social movements and political agendas. The virus response places constraints on Brexit negotiation, green renewal, inequality reduction, the Black Lives Matter movement and so on. In turn, all of these reforms influence the way in which COVID-19 interventions are implemented.

PPE: personal protective equipment.

I have been free with the scattering of etceteras in these columns, signifying that (1) each and every illustration could be pursued in much more detail and (2) there are scores of parallel examples within each category. Hopefully, enough is said to unlock the breadth of the challenge facing epidemic modelling. Can total system change be predicted? Is it possible to build a virtual copy of an open system, which imports and exports influences to and from the wider environment? Is it possible to model unintended consequences? Is it possible to predict social behaviour when predictions may change behaviour and become self-fulfilling or self-denying? Is it possible to anticipate the influence of a programme on a system that continually transforms of its own accord?

The research community remains divided on answers to these questions. A firm ‘yes’ would come from the modelling community, arguing that it is possible to make sound probabilistic estimates of the subjectivities and transformations listed in Box 1. A resolute ‘no’ would come from evaluators and systems analysts who would argue that total comprehension of this system is impossible, with the significant rider that partial knowledge of component processes is quite possible and immensely useful. Interestingly, both paradigms now sit side by side in Westminster. Modellers occupy the box seat in the Scientific Advisory Group for Emergencies (SAGE). Complexity perspectives have gained ground in Defra, Transport and the Treasury. But I utter no further on the fate of the paradigm wars. What can usefully be pondered, however, is Box’s question. Are the specific assumptions built into specific applications used during the COVID-19 crisis good enough to predict the dynamics of the epidemic and the effectiveness of the response?

## Two UK case studies

Much of the UK policy on the epidemic has been informed by models provided by the Imperial College COVID-19 Response Team. In this section, I scrutinise in detail the specific assumptions that guided the construction of two influential simulations – one built before the epidemic had taken hold and one when ‘lockdowns’ had started to be lifted. The first attempted to compare and predict the likely impact of a range of public health measures (termed ‘non-pharmaceutical interventions’) in reducing COVID-19 mortality and the demand for critical care (Ferguson et al., 2020). The second attempted to estimate the effectiveness of NPIs across 11 European countries, and includes a calculation on the total number of deaths averted (Flaxman et al., 2020a, 2020b).

### Report 1: Impact of NPIs to reduce COVID-19 mortality and healthcare demand

**Figure 2.**
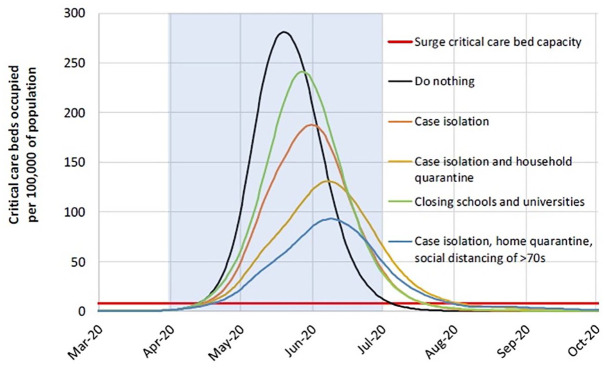
Modelling scenarios. Mitigation strategy scenarios for Great Britain showing critical care (ICU) bed requirements. The black line shows the unmitigated epidemic; the green line shows a mitigation strategy incorporating closure of schools and universities; orange line shows case isolation and household quarantine; and the blue line shows case isolation, home quarantine and social distancing of those over 70. The blue shading shows the 3-month period in which these interventions are assumed to remain in place.

This report was published by the Imperial College Team in March 2020. Figure 2, reproduced from the paper, examines a particular outcome, critical care bed provision required over time, mapped according to which prevention strategy might put into place. The figure follows the ‘without-and-with interventions’ format described in Figure 1. The results are dramatic – different interventions (case isolation, household quarantine, closing schools and hospitals) are predicted to produce significant shifts in both peak capacity requirements for critical care and remissions in the urgency of response.

At face value, these results have clear action implications and immense policy significance (not to mention great visual elegance). One can see at a glance how the curve may be flattened. But are its underlying estimates warranted or mere guesswork? As Box advises the basic suppositions in a model are sometimes implied and sometimes clearly stated, but we can make some headway with a close reading of what is assumed in this particular simulation. The report begins with a detailed catalogue of epidemiological estimates built into the COVID-19 transmission model and then moves on to provide rather scant descriptions of its sociological assumptions about how various NPIs would be implemented and heeded.

Let us begin with the former. Estimates are noted of parameters for population density, household distributions, workplace sizes, commuting distances, school class sizes and so on, used to model person-to-person contact rates. Estimates of the reproduction number (*R*_0_), incubation periods, numbers of symptomatic and asymptomatic individuals, and so on are ascertained from the initial Wuhan outbreak and from other epidemics. Estimates of disease progression in terms of treatment and bed requirements, the number of cases requiring hospitalisation, the number of patients and time needed in intensive care units (ICUs), the number of eventuating deaths are also provided. Some of these metrics are attributed to ‘expert opinion’ and some to ‘personal communication’, for example: ‘We assume that 30% of cases requiring hospitalisation will require critical care (invasive mechanical ventilation or ECMO) based on early COVID-19 cases in the UK, and Italy (Professor Nicholas Hart, personal communication)’ (Ferguson et al., 2020: 5). As can be seen from this brief summary, an immense research effort goes into parameterising the key clinical drivers of the infection. We return to their reliability and validity in due course.

The next batch of estimators is applied to the ‘non-pharmaceutical intervention scenarios’. A number of such action plans are modelled: case isolation, voluntary home quarantine, social distancing as applied to different groups, stopping mass gatherings and the closure of schools and universities. Recall that the models were published in advance of significant government action (and indeed were influential in decision making). This is territory open to all the uncertainties of social complexity. But it is met with estimates than are conspicuously fewer and simpler than in their clinical equivalents and are clearly rooted in the authors’ own assumptions. For instance, the model for an intervention involving the closure of schools and universities assumes: ‘The closure of all schools, 25% of universities remain open. Household contact rates for student families increase by 50% during closure. Contacts in the community increase by 25% during closure’. The model for social distancing those over 70 assumes: ‘Reduce contacts by 50% in the workplace, increase household contacts by 25% and reduce other contacts by 75%. Assume 75% compliance with the policy’.

Both clinical and behavioural assumptions are then loaded into a model and outcomes for a range of different interventions are simulated. Figure 2 depicts some key predictions as they pertain to critical care capacity. Do these inputs warrant the outputs? Are these assumptions sufficiently robust to guide future policy? We are back with Box and indeed Babbage – ‘if you put into the machine wrong figures, will the right answers come out?’

Let us begin with the ‘basic reproduction number’ (*R*_0_). This provides a measure of its transmission potential, the average number of secondary cases generated by a single infected individual. As noted, it is the first ingredient of all models of disease progression – the higher the *R*_0_, the faster the disease will seed. Quite remarkably, there is no agreed figure for the COVID-19 *R*_0_. Many studies have attempted to estimate the basic reproduction number and two overviews of those investigations have collated the various estimates. Its range is reported to vary from 1.5 to 6.49, with a mean of 4.2 in a study by Liu et al. (2020) and from 1.9 to 6.49, with a mean of 3.38 in a review by Alimohamadi et al. (2020). These figures differ from the estimates (mean = 1.4–2.5) widely used by the World Health Organization (2019) and the *R*_0_ of 2.4 used in the Imperial model.

Why the discrepancies? Although the basic definition, ‘the average number of secondary cases generated by an infected individual’ seems impeccably clear, its operationalisation is beset with difficulties. It turns out that there are several different methods for calculating *R*_0_ – starting with the distinction between ‘individual-level’ and ‘population-level’ approaches (Aronson et al., 2020). The former uses real-time contract tracing to follow infection from a diagnosed individual, but the considerable resources required to do this are rarely in place at the outbreak of an infection. The latter, of which there are many variants, *estimates* the *R*_0_ based on different subsets of biological, socio-behavioural and environmental parameters. We note that disputes continue and remain unresolved on the optimal *R*_0_ estimation methods (Breban et al., 2007; Delamater et al., 2019). They need not detain us here, however, for there is a simpler methodological message to deliver – the reproduction number is the first and key ingredient of mathematical models of disease transmission. Its value is itself an estimate, a model within a model. Its contested nature warns us that it is also a seed of the eventual indeterminacy of simulation exercises.^1^

Next, we turn to the model’s assumptions about the impact of the outbreak on health infrastructure. The Imperial simulation centres on ICU provision, so it is appropriate to return to a key driver of the model, namely, the assumption, care of Prof Hart, that ‘30% of cases requiring hospitalisation will require critical care’. To what extent do other inquiries agree with this estimate? There is a considerable discrepancy with real-time figures in the initial UK tracking exercises. Of 16,749 patients with confirmed COVID-19 admitted initially to 166 UK hospitals, ‘17% required admission to High Dependency or Intensive Care Units’ (Docherty et al., 2020). Paradoxically, these authors refer to this comparatively modest figure as a ‘high proportion of patients’. A study from Italy reports, ‘the percentage of patients in intensive care reported daily between 1 March and 11 March 2020 has constantly been between 9% and 11% who are actively infected’ (Remuzzi and Remuzzi, 2020). Another influential survey from the World Health Organization (2020) estimates the surge in ICU requirements as follows:

The data from China suggest that 15–20% of COVID-19 cases require hospitalization, with around 15% of cases presenting with severe symptoms and 5% requiring intensive care. In Italy and Spain, 40–55% of COVID-19 positive cases have been hospitalized, with 7–12% requiring admission to intensive care units.

These figures are derived in (slightly) different time periods, for different countries, using different baselines, but none of the information suggests anything near a definitive, 30 per cent metric for UK hospitalised cases requiring critical care provision. All that can be said with any authority turns out to be a mere truism – the higher the assumed throughput, the more ominous will be the predictions about pressures on critical care capacity, on its ability to cope and on eventual ICU death rates. The Imperial estimate is at the highest end of the range, with obvious and dramatic consequences.

Before we leave the matter, however, there is a much more instructive lesson to be learned from current ICU research. Most mathematical models utilise fixed estimates, as illustrated above. But what is estimated, using a complexity lens, is a responsive, self-transforming process. In model world, it is perceived that there is some enduring context called ICU provision, which will come under arithmetic pressure as the number of cases requiring critical care multiply. In the real world, ICU services are subject to ‘continuous improvement’. Change management is the norm. And crucially from this perspective, ICUs turn from a ‘recipient of’ to a ‘response to’ the virus. They become active interventions rather than passive incumbents.

Phua et al.’s (2020) paper pulls together an extensive catalogue of how ICU practitioners and administrators adapted to and overcame the initial surge in critical care needs. Recommendations are offered on how to improve access and triage, infection protection in the unit, the donning and doffing of personal protective equipment (PPE), the ventilation of units, the spacing of beds, logistical planning for equipment, consumables and pharmaceuticals, workforce load and augmentation, workforce communication and support, patient medication, collecting respiratory tract samples, post-ICU care and so on. The following quotation on ‘intubation’ provides detail on how just one aspect of ICU clinical response can be improved. Non-specialists will find the details obscure; the point is to demonstrate the inevitable and perpetual adaptation of the service predicted by complexity theory but lost in the models.

Intubation of patients with COVID-19 also poses a risk of viral transmission to healthcare workers, and intubation drills are crucial. The most skilled operator available should perform the task with full personal protective equipment (PPE) and the necessary preparation for difficult airways. The number of assistants should be limited to reduce exposure. Bagmask ventilation, which generates aerosols, should be minimised by prolonged pre-oxygenation; a viral filter can be placed between the exhalation valve and the mask. Rapid sequence induction with muscle relaxants will reduce coughing. End-tidal carbon dioxide detection and observation of chest rise should be used to confirm endotracheal tube placement. The use of closed suctioning systems post-intubation will reduce aerosolisation. (Phua et al., 2020)

The same lesson about perpetual adaptation applies to the Imperial modelling of the public health measures that carried the first-wave response to the virus – hygiene, distancing, lockdowns, isolation and so on. Recall the scenarios for school shutdown – ‘The closure of all schools, 25% of universities remain open. Household contact rates for student families increase by 50% during closure’. This sounds simple but is it simplistic? What is really involved? To get a proper measure, it is useful to consult government guidance in the Department for Education (DfE, 2020a) document: ‘Actions for schools during the coronavirus outbreak’. The initial decision is on the management of closure and here we see the first glimmer of complexity in the list of responsible bodies: ‘local authorities, local-authority-maintained schools, academies, free schools, alternative provision schools, pupil referral units, special schools, and independent schools’. The governance of UK schooling is deliberately dispersed, and these bodies have different powers and responsibilities. Rather than imagining there is parametric uniformity to closure, note the report’s very first line of advice: that the guidance should be applied *flexibly* – ‘according to local need’.

The document then moves on to pages and pages of advice of how to implement closure. These involve hundreds of separate recommendations on matters such staffing levels, their pay and protection, attendance expectations and parental liaison, opening hours, curriculum, meals, exams, pooled provision and so on that defy detailed description here. However, what becomes clearer in delving into closure-in-practice is its difference with closure-as-modelled. The simulation operates on the bald assumption: ‘the closure of all schools’. But in reality, most schools remained open – to the ‘children of critical workers’ and to ‘vulnerable children’.

Decisions on who precisely qualified under these criteria and hence on the numbers of residual attendees proved difficult to pin down. A list of eight categories of key workers was provided: (1) Health and social care, (2) Education and childcare, (3) Key public services, (4) Local and national government, (5) Food and other necessary goods, (6) Public safety and national security, (7) Transport, (8) Utilities, communication and financial services. Understandably, these rather abstract categories were subject to continuous clarification – one of which, too ironic to ignore, was that ‘parliamentarians’ are indeed critical workers. But as with all social programmes, decision making is delegated down the implementation chain and so individual schools were left to juggle decisions on whether one or both parents were covered by a requisite category and ultimately whether the child turning up at the school gate was genuinely in the ‘critical’ category. The attendance of ‘vulnerable children’ is subject to equally convoluted regulations, the consequences of which are omitted here.

The point of this example is to show that ‘school closure’ takes on myriad forms. It thus has a variable impact on changing rates of social interaction and, most significantly, it will have complex, uneven and unanticipated effects on disease circulation. And this is only the beginning. Recall from Box 1 that exiting social control is just as complex as applying it. When it comes to ‘reopening’, schools are faced with another, equally large barrage of DfE (2020b) instructions – not only on how they might operate all school routines safely and at a distance but how they should interact with the track-and-trace system, with public transport limitations and so on. Further unknowns accumulate. Some of these re-adaptations may help to diminish the spread of virus but others may escalate its circulation. We arrive at the pertinent conclusion. A proper assessment of how ‘school closure’ assists in controlling the spread of the virus requires an understanding of the workings of all the subprocesses noted in this illustration, which itself is highly abbreviated. It is a classic example of the perils, indeed the impossibility, of trying to capture a complex, self-transforming process as a model ‘parameter’.

### Report 2: Estimating the effects of NPIs on COVID-19 in Europe

A later report by members of the Imperial team attempted to model the ongoing effectiveness of five NPIs (self-isolation, social distancing, banning public events, school closure and lockdown) across 11 European countries (Austria, Belgium, Denmark, France, Germany, Italy, Norway, Spain, Sweden, Switzerland and the United Kingdom). The paper was published in-house as Imperial College COVID-19 Report 13 (Flaxman et al., 2020a) and then summarised in *Nature* in May 2020 (Flaxman et al., 2020b). Given the stature of that journal, the report received immediate international attention. Two of its findings received particular acclaim: (1) ‘We find that, across 11 countries, since the beginning of the epidemic, 3,100,000 [2,800,000–3,500,000] deaths have been averted due to interventions’, (2) ‘Lockdown has an identifiable large impact on transmission (81% [75% - 87%]). The close spacing of interventions in time mean that the individual effects of other interventions are not identifiable’. The public circulation of these findings was immediate. By June 2020, the paper had been accessed 250,000 times with 5000 associated tweets. Verstraeten (2020) reports that the article was used by the Belgian state in a legal procedure ‘to justify the Covid-19 policy of the government stating that this policy has saved 120,000 lives in Belgium’. Various media outlets celebrated the two key conclusions, using versions of the headlines, ‘lockdowns work’, ‘millions of lives saved’ and so on.

The modellers’ task here is challenging – to map *R_t_* and death rates in a specific time period across 11 nations and to attribute the various changes to the national responses, separating out the respective contributions of the five NPIs put in place. Given the timing of this report, more real-time data on the progress of the disease were available, but the basic findings above remain modelled – using a counterfactual comparison between deaths predicted under the five interventions and that estimated as if no interventions had taken place. Achieving this comparison relies, as ever, on the assumptions inserted into the model, which brings us back relentlessly to Box’s test – are they good enough to support the empirical conclusions noted above?

I begin this test by quoting two sociological assumptions built into the Flaxman model:

1. ‘Our parametric form of *R_t_* assumes that changes in the reproduction number are an immediate response to interventions rather than gradual changes in behaviour and does not include importation or subnational variation’.
2. ‘We make the strong assumption that individual interventions have a similar impact in different countries and that the efficacy of those interventions remains constant over time’.

These are strong assumptions indeed but woefully misleading, the problem being that they regard interventions, lockdowns in this instance, as fixed treatments in fixed applications having fixed effects. This contrasts starkly with basic assumption of modern evaluation research, namely, that exploring whether an intervention ‘works’ requires detailed knowledge of (1) its underlying mechanisms, (2) its implementation, (3) its context and (4) its sustainability. In the remainder of this section, I analyse lockdown through these lenses.

#### Lockdown mechanisms

It is useful to start with an understanding of *how* interventions work – to examine what a clinician might term the ‘mechanism of action’ or an evaluator might describe as the ‘programme theory’ of lockdown. In the present case, we begin with a relatively simple and perfectly plausible idea – lockdowns work by restricting social circulation, which in turn reduces person-to-person disease transmission. Alas, this unchallengeable understanding of how lockdowns work raises our first methodological challenge because, of course, the other four interventions in the Flaxman model rest on exactly the same mechanism – and so do other initiatives not included in the model such as border controls, overseas travel bans, post-test quarantine, sporting event biobubbles and so on.

Lockdown, of course, differs from the others in the specifics of how it restricts social circulation, basically by introducing forms of home confinement. In all cases (except Sweden), lockdown came last in the response armoury, with the significant consequence that the other four measures implemented beforehand are retained through lockdown. Thus, in the real world, lockdown is experienced as a composite of a range of ‘social segregation’ interventions. These measures interact – a population familiar with and adapting to social distancing, multiple closures and event bans is thus already primed to ‘stay at home’. Schools, for instance, reported a significant decline in attendance before the formal closure was implemented. It is no surprise then that the compound is more powerful than any of the components but their interconnectivity, their mutuality makes the arithmetical assignment of their relative contributions highly problematic – a bit like looking for success in terms of team membership rather than teamwork. None of these intersections come under consideration in model world where, to echo a previous phrase, interventions are regarded as fixed and separate treatments with fixed and separate effects.

#### Lockdown implementation

It is a commonplace of evaluation research that a programme title signifies little and that the ‘same’ intervention will be implemented in different ways, in different incarnations, with different outcomes. This possibility becomes a probability when that same intervention is implemented across different national polities, cultures and institutions. Comparative research rests on the assumption that we are comparing like with like and that is the challenge that we now put to Flaxman’s 11-nation study. Remember its strong assumption that ‘individual interventions have a similar impact in different countries’.

The report claims, ‘we have tried to create consistent definitions of all interventions’, but the team relies on what might be termed the popular discourse around the concept – the common-sense understanding issuing from guidance materials and public comment thereupon. A table is appended to the report with a perfunctory summary of the lockdown measures as applied in each country (Flaxman, 2020a – Appendix 8). In the Belgian lockdown, ‘Citizens are required to stay at home except for work and essential journeys. Going outdoors only with household members or 1 friend’. The UK lockdown is described as follows, ‘Gathering of more than 2 people not from the same household are banned and police enforceable’. The French lockdown requires ‘Everybody to stay at home. Need a self-authorisation form to leave home’. The Spanish lockdown is afforded two words ‘Nationwide lockdown’. These descriptions are sourced haphazardly – sometimes from official bodies (Ministero della Salute, Bundesamt fur Gesundheit, etc.) but often from media accounts (Guardian, BBC, Sveriges-Radio, CNN, etc.). Nothing here persuades me that the nature of each lockdown has been explored thoroughly. Nothing ensures that the subsequent analysis goes on to compare like with like.

It would be too massive an undertaking for this article to chart the extensive differences in lockdown implementation in each country, but such efforts exist including a monumental exercise ‘National responses to the COVID-19 pandemic’ in Wikipedia (2020). I can, however, offer a first glimpse of the complexity by summarising the UK lockdown rules. First, note that just like the guidance on school closure noted earlier, formal guidance on lockdown runs to many, many pages (GovUK, 2020a) and that this guidance is withdrawn and replaced periodically (GovUK, 2020b). Moreover, there is separate extensive documentation on ‘enforcing the lockdown laws’ (Commons Library, 2020). Note also that what the model analyses as the ‘UK’ response ignores the devolution of responsibility for public health to the United Kingdom’s devolved administrations of Scotland, Wales and Northern Ireland. Their parliaments have tended to be critical of ‘English’ responses and therefore produced their own separate rulebooks and timetables for lockdown.

Coming to the rules themselves, they cover movement restrictions on shopping, exercise, medical needs, travel to work, funerals, places of worship, social services, mental health provision, hospital and care home visits, house moves, libraries, playgrounds, pubs, cinemas, theatres, restaurants, takeaway services, parks, playgrounds, campsites, public gatherings, construction sites, outdoor businesses and so on. Many items receive supplementary clarification over time such as on the permissible size, purpose and location of ‘outdoor gatherings’. Lockdown, in short, is a lengthy, intricate, evolving business, making a nonsense of the model’s basic assumption that changes in behaviour are an ‘immediate response’ to interventions.

But we have yet to reach the major lacuna in the Flaxman assumptions, which is that each of the 11 countries implemented these regulations in different ways – sometimes subtly and sometimes starkly. Take the initial policing of lockdown in just two countries as an example:

Enforcing the UK lockdown was widely seen by chief constables as ‘impossible’ (Guardian, 2020). The English and Welsh forces thus adopted a ‘policing by consent model’, termed the ‘Four E’s’. (1) *Engage*: Officers speak to people and try to establish their ‘awareness and understanding of the situation’. (2) *Explain*: Officers ‘try to educate people’ about the coronavirus risks. (3) *Encourage*: Officers encourage people to ‘act reasonably’. (4) *Enforce*: Officers may ‘as a last resort, remove a person to the place where they live, using reasonable force only if it is a necessary and proportionate means of ensuring compliance’.

French policing of ‘confinement’ was much more robust, especially for regions designated ‘Red Zones’. The number and types of venues allowed to remaining open were significantly less than in the United Kingdom. Citizens had to carry documentation certifying why they are outside, including personal details, reason, date, reason and precise timing. Tight distance restrictions were imposed for travel that was permitted. Checkpoints were installed across the country. Fines began at €38 but were upped to €135. Repeat offenders faced fines of €1500. In total, 100,000 police were deployed to manage these obligations. They conducted approximately 19 million checks throughout the nation’s 8-week lockdown (Euronews, 2020).

Extend this comparison to a further nine countries and there is little to suggest that lockdowns are ‘similar’, less still that they have a common impact.

#### Lockdown contexts

This brings us to the next lacunae in Flaxman et al.’s model. Rather than assuming that programmes work universally, the norm in contemporary evaluation research is to suppose that they will vary in effectiveness. The crucial imperative is to investigate ‘what works for whom and in what circumstances’ (Pawson and Tilley, 1997). The importance of such contextual constraints become apparent if we think back to the mechanism of action of lockdown – minimise social interaction to reduce disease transmission. To achieve this end requires a close appreciation of pre-existing forms of social interaction, which vary between individuals, communities and countries. We need to understand these networks in order to close them down. But such considerations are generally absent in epidemic modelling and in particular in the strong assumptions of the Flaxman simulation.

‘This ignores one of the most important empirical findings of research on complex social networks during the last twenty years . . . social networks are highly heterogeneous in terms of degree distributions’ (Manzo, 2020). Even within a ‘single’ social group, contacts do not occur randomly; some individuals, some subgroups, some situations will have high levels of connectivity; and some of these will be relatively isolated. This should not come as a surprise to epidemiologists – sexually transmitted disease increases most markedly through individuals with many partners (known as ‘hubs’ or ‘connectors’) rather than through individuals with one or no sexual partners.

In the case of lockdown measures that are modelled nationwide or continent-wide, this finding suggests that the research will not have sufficient levels of ‘granularity’ to recognise the high degree of heterogeneity in the networks of transmission. The same problem, of course, applies to the policies themselves – implemented ‘across the board’ without sufficient attention to the hubs and connectors. If we start with the assumption that connectivity and transmission are deeply nuanced, then it becomes clear that lockdown (contra the models) will have markedly different effectiveness within and between nations. Let us illustrate with a simple example that conveniently begins with the idea of hubs. In the United Kingdom, the epidemic was first identified in London. Its initial high excess mortality can be traced to several factors: it imported infection by being the major hub for international travel; its mass transport system demands close physical contact; high-density living, working and shopping does the same; and hosting major cultural and sporting events also has a herding effect. Note that this configuration pinpoints a particular location in one country as a ‘connector’, but it is an explanation that has the power to differentiate national epidemics. Some *but not all* European countries will have major conurbations that share these characteristics.

By the same token, there are countless other differences in social connectivity that will generate the heterogeneous spread of the disease and account for the inconsistent impact of the lockdown. A recent paper by Aron et al. (2020) begins to identify the many subgroups that harbour different levels of social interaction and that go on to shape local disparities in excess mortality: ethnic group composition, age distribution, family and household composition, obesity and comorbidity levels, poverty and inequality, housing types and condition, numbers in institutions (care home and prisons), cultural practices, as well as significant disparities in healthcare capacity. All of these contexts vary significantly within and between countries. We are not ‘all in this together’. The virus is selective in its impact. Infection is not random. All of this ‘small-world topology’ is overlooked if ‘scientists and policy makers can only think in terms of generalised interventions concerning large and undifferentiated groups of people’ (Manzo, 2020).

#### Lockdown sustainability

Another vital concern of contemporary evaluation research lies with the sustainability of interventions. A key issue for projects in community regeneration, international development, public health, crime reduction and many others can be put as follows – what happens next? What happens when funding stops, when additional resources are withdrawn, when participants are left to their own devices? This dynamic is, of course, of essential interest in virus reduction, where the very idea is to scale down lockdown measure at the point where disease transmission is under control and the impact of the NPIs presumed to be sustainable. This policy premise involves the brave assumption that behavioural changes are sufficiently deep rooted for their impact to endure. People will continue to follow guidance and endure some deprivation for a considerable period in order to enhance the public good. The Flaxman model shares this same conviction – ‘We make the strong assumption that the efficacy of those interventions remains constant over time’.

Is this remotely realistic? There is no intervention in the world that has generated a uniform response from its recipients and we know from media ‘vox pop’ that reactions to the complex restrictions introduced to combat the virus unfold in scores of different ways – some people comply, some resist, some learn, some volunteer support, some grow weary, some change their minds, some seek exceptions and so on. The crucial question in respect of sustainability is whether overall public trust in the management of the epidemic remained ‘constant over time’ and by summer 2020 pertinent evidence began to emerge.

A report for the All-Party Parliamentary Group on Social Integration (2020) charts a pronounced bell shape in support. Initially, lockdown forged a ‘new community spirit’ with a rise in mutual aid, volunteering, donations, tech literacy campaigns and strong public demonstrations in favour of front-line workers. The report then goes on to describe how solidarity began to fray and how a backlash to lockdown mounted due to intervention fatigue, the differential impact of the virus across communities, confusions caused by rule changes and weariness of political infighting.

One trigger of growing resistance to interventions is of particular relevance to the lockdown. It is known as the ‘free rider problem’. Pareto (1935) describes it as follows:

If *all* individuals refrained from doing A, every individual as a member of the community would derive a certain advantage. But now if all individuals less *one* continue refraining from doing A, the community loss is very slight, whereas the one individual doing A makes a personal gain far greater than the loss that he incurs as a member of the community.

In the present case, if one person ignores the lockdown, she or he gains from the collective effort, without having to make an individual contribution. The problem occurs when one becomes two and two becomes many. A sense of injustice amplifies if free riding becomes conspicuous and commonplace, generating division and leading the collective effort to crumble.

Arguably, the activities of free riders had such an effect on UK public trust in the management of the epidemic. A longitudinal survey by Fancourt et al. (2020) charts the changes in public trust in the government handling of the pandemic. Starting on 22 May, there was a steep decrease in confidence, which has never recovered. This date coincides with the discovery that a senior public official had broken lockdown rules with a 500-mile round trip to a family estate. The fact that this official had abstained from collective responsibility ignited a torrent of abuse – ‘one rule for those in charge and one rule for everyone else’. I should add that the lockdown encountered other prominent free riders, including a mathematical biologist and a senior Scottish Health official. After these high-profile incidents, the negative and lasting decline in public confidence was further exacerbated by crowds of anonymous free riders who gathered in parks and beaches in the early summer.

## Conclusion

Box asks us to pursue this question, ‘Is the model good enough for this particular application?’ And on this basis, I have made the case that two influential models are potentially misleading, often arbitrary and clearly self-affirming. The mechanical assumptions and fixed statistical estimates built into the Imperial simulations completely fail to reflect the complexity of the societal response to the COVID-19 interventions. What follows?

Policy makers need to beware of the spurious precision and the seductive visual representation of the transmission curves in epidemic modelling. They might hark back to advice from an inquiry on UK governmental actions during the 2009 influenza pandemic: ‘modellers are not court astrologers’ (Hine, 2010).

Evidence on how to contain the virus must be drawn from a wider range of inquiries, pursuing a broader range of research methodologies, including process, programme theory and case study evaluations. The evidence fragments used earlier in this article are used critically but, marshalled together with other local, pragmatic research, they have a profoundly positive role in building more adequate explanations of how interventions work.

Methodological pluralism should be reflected in the composition of the UK government’s SAGE. The first-wave response to the virus consists entirely of social interventions and yet modellers and epidemiologists rather than programme evaluators dominate the ranks of senior advisors. It would be wiser to ‘Box and Cox’, the seats in methodological high command.

Research progresses by eschewing certainty and by encouraging debate and competition. All research methods are fallible, and the science of COVID-19 would benefit from greater humility. The profound philosophical and sociological accounts of the privileged status of scientific knowledge recognise that it owes that status through ‘organised scepticism’ (Merton, 1968) and close mutual monitoring from the ‘disputations community of truth seekers’ (Campbell, 1988).

UK policy making has preferred evidence with a macro-focus, generating a stuttering stream of national restrictions (lockdowns, circuit breaking, rule-of-six, etc.) based on overzealous extrapolations of shifts in aggregate, national data. There should be more emphasis on the micro-circuits of transmission, seeking and targeting continuous quality improvement in the many subprocesses, logistics and agencies that embody the everyday response to the virus.

Administrative science has studied government decision making under conditions of duress for many a year and maintains a thread running back to a paper by Lindblom (1959), with the unprepossessing title: ‘The science of muddling through’. What this body of work suggests is the avoidance of misplaced certainty and its replacement by adaptive policy and devolved decision making. Instead of trying to suppress the virus, it may be time to learn to live with it and to concentrate on managing, minimising and balancing risks.

## Notes

1. The *R* number is also used to chart the progression of the disease over time, where it is referred to as the *effective* reproduction number (denoted *R_t_*). An *R_t_* of 1 signifies that the total number of new infections is stable. ‘Keeping the *R* number below 1’ thus became an unlikely political mantra and indeed a formal performance indicator of the effectiveness of the government response. Again, some major caveats are appropriate. Most significantly, *R_t_* remains estimated (and thus contested) rather than directly observed. It is highly context-sensitive, contact networks and *R_t_* always varies significantly from location to location, community to community. It is also highly time sensitive, and will increase and decrease naturally in the absence of formal interventions.

## References

[bibr1-1356389020968579] Adam D (2020) Modelling the pandemic: The simulations driving the world’s response to COVID-19. Nature 580(16): 316–318. 32242115 10.1038/d41586-020-01003-6

[bibr2-1356389020968579] Alimohamad Y Taghdir M Sepandi M (2020) The estimate of the basic reproduction number for novel coronavirus disease (COVID-19): A systematic review and meta-analysis. Available at: https://www.jpmph.org/upload/pdf/jpmph-20-076.pdf 10.3961/jpmph.20.076 PMC7280807 32498136

[bibr3-1356389020968579] All-Party Parliamentary Group on Social Integration (2020) Social connection in the COVID-19 crisis. Available at: http://www.britishfuture.org/wp-content/uploads/2020/05/Social-Connection-in-the-COVID-19-Crisis.pdf

[bibr4-1356389020968579] Aron J Giattino C Muellbauer J, et al (2020) A pandemic primer on excess mortality statistics and their comparability across countries. Available at: https://www.inet.ox.ac.uk/files/17.10-29-Jun-20-Aron-Muellbauer-Giattino-Ritchie-Excess-Mortality-article.pdf

[bibr5-1356389020968579] Aronson J Brassey J Mantai K (2020) When will it be over? An introduction to viral reproduction numbers, R0 and Re. Available at: https://www.cebm.net/covid-19/when-will-it-be-over-an-introduction-to-viral-reproduction-numbers-r0-and-re/

[bibr6-1356389020968579] Babbage C (1864) Passages from the Life of a Philosopher. Longman: London.

[bibr7-1356389020968579] Box G (1976) Science and statistics. Journal of the American Statistical Association 71(356): 791–799.

[bibr8-1356389020968579] Box G Luceño A (1998) Statistical Control: By Monitoring and Feedback Adjustment. New York: John Wiley & Sons.

[bibr9-1356389020968579] Breban R Vardavas R Blower S (2007) Theory versus data: How to calculate R0? PLoS ONE 2(3): e282. 17356693 10.1371/journal.pone.0000282 PMC1804098

[bibr10-1356389020968579] Campbell D (1988) Methodology and Epistemology for the Social Sciences. Chicago, IL: University of Chicago Press.

[bibr11-1356389020968579] CECAN (Centre for the Evaluation of Complexity Across the Nexus) (2018) Policy evaluation for a complex worlds. Available at: https://www.cecan.ac.uk/resources

[bibr12-1356389020968579] Commons Library UK (2020) Available at: https://commonslibrary.parliament.uk/research-briefings/cbp-8875/

[bibr13-1356389020968579] Delamater PL Street EJ Leslie TF, et al (2019) Complexity of the basic reproduction number (R0). Emerging Infectious Diseases 25(1): 1–4. 10.3201/eid2501.171901 PMC6302597 30560777

[bibr14-1356389020968579] Department for Education (DfE) (2020a) Actions for schools during the coronavirus outbreak: What schools need to do during the coronavirus (COVID-19) outbreak. 22 3 Available at: https://www.gov.uk/government/publications/covid-19-school-closures

[bibr15-1356389020968579] Department for Education (DfE) (2020b) Guidance for full opening of schools. Available at: https://www.gov.uk/government/publications/actions-for-schools-during-the-coronavirus-outbreak/guidance-for-full-opening-schools

[bibr16-1356389020968579] Docherty A Harrison E Green C, et al (2020) Features of 16,749 hospitalised UK patients with COVID-19 using the ISARIC WHO clinical characterisation protocol. medRxiv. Available at: https://www.medrxiv.org/content/10.1101/2020.04.23.20076042v1 10.1136/bmj.m1985 PMC7243036 32444460

[bibr17-1356389020968579] Euronews (2020) Available at: https://www.euronews.com/2020/05/07/amnesty-slams-alleged-police-brutality-in-french-lockdown-enforcement

[bibr18-1356389020968579] Fancourt D Steptoe A Wright L (2020) The Cummings effect: Politics, trust, and behaviours during the COVID-19 pandemic. The Lancet. Epub ahead of print 6 August. DOI: 10.1016/S0140-6736(20)31690-1. PMC7613216 32771083

[bibr19-1356389020968579] Ferguson N Laydon D Nedjati-Gilani G, et al (2020) Report 9: Impact of non-pharmaceutical interventions (NPIs) to reduce COVID-19 mortality and healthcare demand. Available at: https://www.imperial.ac.uk/media/imperial-college/medicine/sph/ide/gida-fellowships/Imperial-College-COVID19-NPI-modelling-16-03-2020.pdf 10.1007/s11538-020-00726-x PMC7140590 32270376

[bibr20-1356389020968579] Flaxman S Mishra S Gandy A, et al (2020a) Estimating the effects of non-pharmaceutical interventions on COVID-19 in Europe. Available at: https://www.imperial.ac.uk/media/imperial-college/medicine/mrc-gida/2020-03-30-COVID19-Report-13.pdf 10.1038/s41586-020-2405-7 32512579

[bibr21-1356389020968579] Flaxman S Mishra S Gandy A, et al (2020b) Estimating the effects of non-pharmaceutical interventions on COVID-19 in Europe. Nature 584: 257–261. 32512579 10.1038/s41586-020-2405-7

[bibr22-1356389020968579] Gates EF (2016) Making sense of the emerging conversation in evaluation about systems thinking and complexity science. Evaluation and Program Planning 59: 62–73. 27591941 10.1016/j.evalprogplan.2016.08.004

[bibr23-1356389020968579] Gerrits L Verweij S (2015) Taking stock of complexity in evaluation: A discussion of three recent publications. Evaluation 21(4): 481–491.

[bibr24-1356389020968579] GovUK (2020a) Available at: https://www.gov.uk/government/publications/full-guidance-on-staying-at-home-and-away-from-others/full-guidance-on-staying-at-home-and-away-from-others

[bibr25-1356389020968579] GovUK (2020b) Available at: https://www.gov.uk/government/publications/coronavirus-outbreak-faqs-what-you-can-and-cant-do/coronavirus-outbreak-faqs-what-you-can-and-cant-do

[bibr26-1356389020968579] Guardian (2020) Available at: https://www.theguardian.com/global/2020/mar/24/police-leaders-say-enforcing-uk-lockdown-may-be-impossible

[bibr27-1356389020968579] Hine D (2010) The 2009 Influenza Pandemic: An Independent Review of the UK Response to the 2009 Pandemic. London: Cabinet Office.

[bibr28-1356389020968579] HM Treasury (2020) The Magenta Book: HM Treasury guidance on what to consider when designing an evaluation. https://www.gov.uk/government/publications/the-magenta-book

[bibr29-1356389020968579] Keeling M Danon L (2009) Mathematical modelling of infectious diseases. British Medical Bulletin 92: 33–42. 19855103 10.1093/bmb/ldp038

[bibr30-1356389020968579] Lindblom C (1959) The science of ‘muddling through’. Public Administration Review 19(2): 79–88.

[bibr31-1356389020968579] Liu Y Albert A Gayle A, et al (2020) The reproductive number of COVID-19 is higher compared to SARS coronavirus. Journal of Travel Medicine 27(2): taaa021. 32052846 10.1093/jtm/taaa021 PMC7074654

[bibr32-1356389020968579] Manzo G (2020) Complex social networks are missing in the dominant COVID-19 epidemic models. Sociologica 14(1): 31–49.

[bibr33-1356389020968579] Merton R (1968) Social Theory and Social Structure. New York: Free Press.

[bibr34-1356389020968579] Pareto V (1935) The Mind and Society. New York: Harcourt Brace.

[bibr35-1356389020968579] Pawson R Tilley N (1997) Realistic Evaluation. London: SAGE.

[bibr36-1356389020968579] Phua J Weng L Ling L, et al (2020) Intensive care management of coronavirus disease 2019 (COVID-19): Challenges and recommendations. The Lancet Respiratory Medicine. Epub ahead of print 6 April. DOI: 10.1016/S2213-2600(20)30161-2. PMC7198848 32272080

[bibr37-1356389020968579] Remuzzi A Remuzzi G (2020) COVID-19 and Italy: What next? Lancet Health Policy 395(10231): 1225–1228. Available at: https://www.thelancet.com/journals/lancet/article/PIIS0140-6736(20)30627-9/fulltext 10.1016/S0140-6736(20)30627-9 PMC7102589 32178769

[bibr38-1356389020968579] Siettos C Russo L (2013) Mathematical modelling of infectious disease dynamics. Virulence 4(4): 295–306. 23552814 10.4161/viru.24041 PMC3710332

[bibr39-1356389020968579] Verstraeten M (2020) Comment on Flaxman. Available at: https://www.nature.com/articles/s41586-020-2405-7#article-comments

[bibr40-1356389020968579] Wikipedia (2020) Available at: https://en.wikipedia.org/wiki/National_responses_to_the_COVID-19_pandemic

[bibr41-1356389020968579] World Health Organization (2019) Coronavirus disease 2019 (COVID-19) situation report – 46. Available at: https://www.who.int/docs/default-source/coronaviruse/situation-reports/20200306-sitrep-46-covid-19.pdf?sfvrsn=96b04adf_4

[bibr42-1356389020968579] World Health Organization (2020) Health systems respond to COVID-19 technical guidance #2, creating surge capacity for acute intensive care recommendations for the WHO and European region. Available at: http://www.euro.who.int/__data/assets/pdf_file/0006/437469/TG2-CreatingSurgeAcuteICUcapacity-eng.pdf

